# Harnessing Inflammation Resolution in Arthritis: Current Understanding of Specialized Pro-resolving Lipid Mediators’ Contribution to Arthritis Physiopathology and Future Perspectives

**DOI:** 10.3389/fphys.2021.729134

**Published:** 2021-09-01

**Authors:** Tiago H. Zaninelli, Victor Fattori, Waldiceu A. Verri

**Affiliations:** ^1^Laboratory of Pain, Inflammation, Neuropathy, and Cancer, Department of Pathology, Londrina State University, Londrina, Brazil; ^2^Vascular Biology Program, Boston Children’s Hospital, Department of Surgery, Harvard Medical School, Boston, MA, United States

**Keywords:** rheumatic diseases, DMARDs, SPMs, rheumatoid arthritis, osteoarthritis

## Abstract

The concept behind the resolution of inflammation has changed in the past decades from a passive to an active process, which reflects in novel avenues to understand and control inflammation-driven diseases. The time-dependent and active process of resolution phase is orchestrated by the endogenous biosynthesis of specialized pro-resolving lipid mediators (SPMs). Inflammation and its resolution are two forces in rheumatic diseases that affect millions of people worldwide with pain as the most common experienced symptom. The pathophysiological role of SPMs in arthritis has been demonstrated in pre-clinical and clinical studies (no clinical trials yet), which highlight their active orchestration of disease control. The endogenous roles of SPMs also give rise to the opportunity of envisaging these molecules as novel candidates to improve the life quality of rhematic diseases patients. Herein, we discuss the current understanding of SPMs endogenous roles in arthritis as pro-resolutive, protective, and immunoresolvent lipids.

## Introduction

Rheumatic diseases, represented by varied forms of arthritis and other musculoskeletal disorders, affect millions of people around the world. Rheumatoid arthritis (RA), osteoarthritis (OA), septic arthritis, and gouty arthritis are some examples of this painful group of diseases (Gourley and Miller, 2007) and the focus of this review. Historically, pain in rheumatic disease is mostly attributed to tissue damage mainly due to neutrophil recruitment during the active phase of the diseases (Fattori et al., 2016). These immune cells are equipped with a vast arsenal of molecules that are released with the aim of protecting the host during infections, for example, but at the same time cause inflammation and pain. The release of pro-inflammatory cytokines such as TNF-α, IL-1β, IL-33, and the process of NETosis are widely known to aggravate arthritis disease status and pain (Fattori et al., 2016). Recent evidence also suggests that antibody immunocomplex activates nociceptors. By acting on FcγRI and FcγRIIb receptors expressed by mouse TRPV1^+^ dorsal root ganglion (DRG) neurons, these immunocomplexes induce the release of neuropeptide and activation of nociceptors to produce pain (Bersellini Farinotti et al., 2019).

Arthritis, in their different forms, are traditionally regarded as a life-long disease. As for diabetes, hypertension, and certain forms of cancer, current therapies for arthritis focus on disease control as cure still seems out of reach. Therefore, life-long treatment to control the inflammatory process is required to effectively prevent further cartilage and bone destruction. To the date, disease-modifying anti-rheumatic drugs (DMARDs) are one the current choice (alone or in combination) for the treatment of different types of arthritis (Singh et al., 2016; Kolasinski et al., 2020). DMARDs can be categorized into conventional synthetic (cs) DMARDs (e.g., methotrexate), biologic (b) DMARDs (anakinra, etanercept) and, most recently introduced, targeted synthetic (ts) DMARDs [Janus Kinase inhibitor] (De Cock and Hyrich, 2018). While many patients experience good disease control with DMARDs, a fraction of patients continues to experience significant pain even with low disease activity (Welsing et al., 2005; Lee et al., 2014) or in remission (Lee et al., 2011). In addition, typical side effects such as increase susceptibility to infections, development of adaptive immunity against the biological agents (Fattori et al., 2016; Singh et al., 2016; Kolasinski et al., 2020), relapse of active disease, and the concomitant increase of joint pain are not uncommon (Schipper et al., 2010). Infectious arthritis are treated with a combination of broad-spectrum antibiotic with corticosteroids, immunobiological agents, or opioids for pain management, which might facilitate pathogen spread due to immunosuppression (Orlicka et al., 2013; Cabral et al., 2016; Plein and Rittner, 2018). The high cost and wide range of side effects of these drugs frequently restricts their usage, which highlights this unmet need of new compounds to treat arthritis.

Exogenous administration of different specialized pro-resolving lipid mediators (SPMs) at low doses has been shown effectiveness at treating pain and infection in experimental models (Buckley et al., 2014; Serhan, 2017; Fattori et al., 2020). In oppose to the current clinically active drugs for arthritis treatment, SPMs present long lasting analgesic and anti-inflammatory effects and are not immunosuppressive compounds (Buckley et al., 2014; Serhan, 2017; Fattori et al., 2020). This characteristic of SPMs of blocking pain without immunosuppression rendered the term “immunoresolvent” to this class of molecules. Therefore, we review pre-clinical and clinical data involving SPMs in arthritis as well as the potential outcomes of this knowledge to arthritis therapeutics.

### Specialized Pro-Resolving Lipid Mediators and Resolution of Inflammation

While it is commonly attributed to Hippocrates the use of willow bark to treat the signs of inflammation around 400 Before Common Era (BCE), it is known that the use of willow extracts dates from around 4,000 BCE by ancient civilization such as the Assyrians (Mahdi, 2010). From then, to the total organic synthesis of salicylic acid (1850s), and later to the acetylation of salicylic acid (1900s), aspirin remains one of the most used drugs (Mahdi, 2010). Part of that is attributed to the seminal discoveries of Prof Sergio H. Ferreira and Sir John R. Vane in the field of pharmacology by showing how aspirin works and why it reduces inflammation and inflammatory pain (Ferreira et al., 1971; Vane, 1971; Ferreira, 1972). Drug discovery to treat inflammation and pain, therefore, mainly focused on mimicking aspirin mechanism of action giving rise to the COX-blockers. However, COX-2 acetylation by aspirin changes the activity of this enzyme leading to the production of SPMs, which are responsible, in part, for the anti-inflammatory and analgesic mechanisms of aspirin. This knowledge makes clear now that stimulation of endogenous pathways involved in the resolution of inflammation can lead to a new road for drug discovery.

The resolution of inflammation is controlled by a time-dependent mechanism of SPM production (Levy et al., 2001; Bannenberg et al., 2005). A seminal study using an air-pouch model of inflammation induced by TNF-α shows the occurrence of a biosynthetic shift from pro-inflammatory to pro-resolving lipid mediators (Levy et al., 2001). This work demonstrates that an increase of lipoxin A_4_ (LXA_4_) levels correlates with the reduction in PGE_2_ production, neutrophil recruitment, and consequently, the resolution of inflammation (Levy et al., 2001). SPMs are divided into four main families: the LX, the maresin (MaR), the resolvin (Rv), and the protectin (PD; Serhan et al., 2015; Chiang and Serhan, 2017; Serhan, 2017). Endogenous biosynthesis of SPMs depends on the action of different enzymes to convert arachidonic acid (AA), eicosapentaenoic acid (EPA), docosapentaenoic acid (DPA), or docosahexaenoic acid (DHA) into distinct molecules within the different SPM classes (Buckley et al., 2014; Serhan, 2017; Fattori et al., 2020). The biological effects of SPMs are receptor-dependent and occur via the activation of specific G protein-couple receptors. The receptors and expressing cellular types are summarized in Table 1. The activity of SPMs has been widely explored since several studies demonstrate these molecules might produce an enduring effect. Treatment with LXA_4_ 72 h before stimulus increases the efficacy of this mediator against skin damage induced by ultraviolet B radiation. For RvD1, when treatment is performed before the development of tactile allodynia, RvD1 produces 30 days of analgesic effect in opposed to its limited analgesic effect with treatment at later time points provide limited analgesia (Huang et al., 2011). MaR1, on the other hand, upon a single treatment, displays 5 days of analgesic effect when treatment is performed before stimulus with CFA and 3 days of analgesia when treatment is performed 24 h after the stimulus (Fattori et al., 2019). In corroboration to our study, Allen and colleagues demonstrated that MaR1 presents 14 days of analgesia after repeated treatments (Allen et al., 2020). These sets of data show that isolated SPMs demonstrate time-dependent and long-term efficacy even upon single treatment, which might be useful for the treatment of inflammatory diseases.

**TABLE 1:** SPMs and their receptors.

SPM family		SPM	Receptor	Cell type	References
Lipoxins		LXA_4_	GPR32 ALX/FPR_2_	**ALX/FPR2** – macrophage, neutrophil, lymphocyte, natural killer, ILC2. **GPR32** – macrophage, neutrophil, and lymphocyte.	Hodges et al., 2017
		AT-LXA4			
Resolvins	D-series	RvD1			Krishnamoorthy et al., 2012
		AT-RvD1			
		RvD2	GPR18	Macrophage, neutrophil, neurons, and astrocytes	Chiang et al., 2015; Zhang et al., 2018
		RvD3	ALX/FPR_2_	Macrophage, neutrophil, lymphocytes, natural killer, ILC2.	Arnardottir et al., 2016
		RvD5	GPR101	Macrophages, neutrophils, and monocytes.	Flak et al., 2020
	E-series	RvE1	ChemR23	Macrophage, dendritic cell, natural killer, ILC2, and neurons.	Herova et al., 2015; Deyama et al., 2018; Motwani et al., 2018
		RvE2			
Protectins		PD1/NPD1	GPR37	Macrophage and neutrophil	Marcheselli et al., 2010; Bang et al., 2018
Maresins		MaR1	LGR6	Macrophage and neutrophil	Chiang et al., 2019

*SPMs, Specialized pro-resolving mediators; LXA4, Lipoxin A4; AT-LXA4, aspirin-triggered lipoxin A4; RvD1, resolvin D1; AT-RvD1, aspirin-triggered resolvin D1; GRP32, G Protein-Coupled Receptor 32; ALX/FPR2, G-protein coupled formyl peptide receptor 2; ILC2, type 2 innate lymphoid cells; RvD2, resolvin D2; GPR18, G Protein-Coupled Receptor 18; RvD3, resolvin D3; RvD5, resolvin D5; GRP101, G Protein-Coupled Receptor 101;RvE1, resolvin E1; RvE2, resolvin E2; ChemR23, Chemerin Receptor 23; PD1, protectin D1; NPD1, neuroprotectin D1; GRP37, G Protein-Coupled Receptor 37; MaR1, maresin 1; and LGR6, leucine rich repeat containing G protein-coupled receptor 6.*

Further studies focusing on the immune cell side of the resolution, show that the initial production of SPMs are followed by the recruitment of a distinct subpopulation of pro-resolving macrophages, which correlates with the resolution of inflammation (Bannenberg et al., 2005). This was demonstrated using the self-resolving model of peritonitis induced by zymosan (1 mg, ip; Bannenberg et al., 2005). Subsequent studies using the same self-resolving model of peritonitis induced by zymosan (but now using 0.1 mg, ip) by Derek Gilroy’s group, shed light on the role and phenotype of these pro-resolving macrophages (Bystrom et al., 2008; Stables et al., 2011; Newson et al., 2014). These cells possess a unique phenotype that is controlled by cAMP while sharing some markers with M1 macrophages such as inducible nitric oxide synthase (iNOS) and COX-2 (Bystrom et al., 2008). Transcriptomic analysis reveals that, while the resolution was also achieved after injection of 10 mg of zymosan, a higher number of M1-like macrophages without the acquisition of the pro-resolving phenotype were generated when compared to stimulus with 0.1 mg of zymosan (Stables et al., 2011). These results might indicate that hyperinflammatory states possibly compromise host response against subsequent injury and complete resolution (Stables et al., 2011). Subsequent analysis 60 days after injection of zymosan (0.1 mg, ip) shows that resolving inflammation changes the immune cell landscape of the peritoneal cavity. This new immune cell landscape provides a more rapid and effective response against secondary tissue injury, indicating the existence of a possible tissue memory mediated by pro-resolving macrophages (Newson et al., 2014). Therefore, in addition to the production of SPMs, complete resolution might be only achieved after this third phase of leukocyte recruitment that is mainly dominated by tissue memory-generating macrophages (Bystrom et al., 2008; Stables et al., 2011; Newson et al., 2014).

## SPM Levels and Arthritis Status

### SPM Levels and Arthritis Status in Humans

**FIGURE 1:**
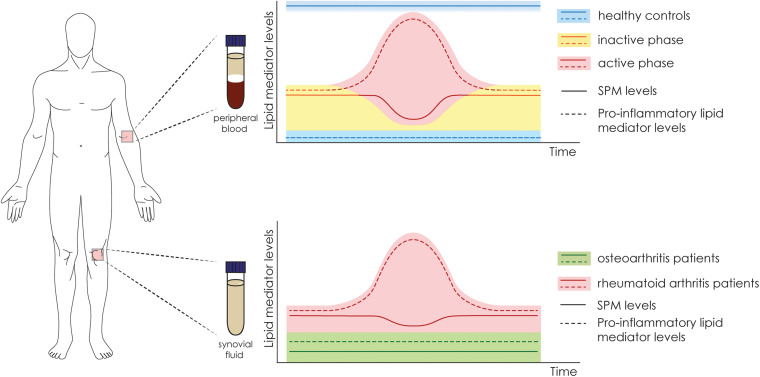
Fluctuation in the levels of SPMs and pro-inflammatory mediators in human samples of serum and synovial fluid. Schematic representation of the fluctuation in the levels of lipid mediators according to the authors’ interpretation. In serum samples of healthy controls (in blue), there are high levels of SPMs and low levels of pro-inflammatory mediators. In arthritic patients, the levels of SPM fluctuate according to the disease phase, which varies between inactive (yellow) and active (red). In the synovial fluid (bottom panel), the levels of SPMs are higher in RA patients (red) in comparison to osteoarthritis patients (green). In contrast, the levels of pro-inflammatory mediators are also higher in RA patients, especially in the active phase.

**FIGURE 2:**
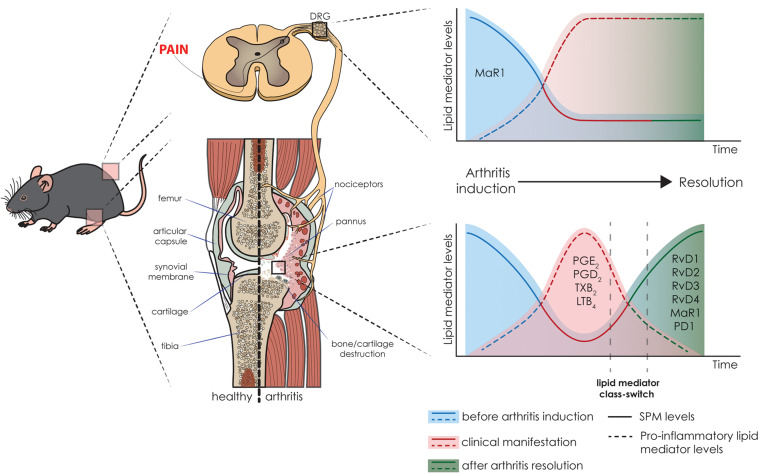
Pre-clinical levels of lipid mediators and their correlation with arthritis status. Schematic representation of the fluctuation in the levels of lipid mediators, i.e., pro-inflammatory lipid mediators and SPMs, in arthritis initiation, resolution, and associated pain in the K/BxN-induced arthritis model. The time-dependent resolution process is singular and tissue-specific; therefore, there is a variation in the resolution between the joint and DRG. In the joint, the inflammatory events lead to an increase in pro-inflammatory lipid mediators and decrease in SPM levels. In the resolution phase, the lipid class switch supports the inflammation resolution and restore the levels of SPMs (Arnardottir et al., 2016). While the joint has the health status restored, in the DRG, the class switch does not occur at the same pace, and the imbalance between pro-inflammatory mediators and SPMs in the dorsal root ganglia (DRG) result in persistent pain (Allen et al., 2020).

In this section, we discuss the profile and role of SPMs in arthritis. Figures 1, 2, and Table 2 summarize our discussion. Despite the common sense of arthritis patients’ abilities to predict weather changes, the balance between pro-inflammatory and SPMs could be closely related to this phenotype. Previous studies have shown that the deficiency of 12/15-lipoxigenase, a key enzyme in the synthesis of SPMs, are related to worsened outcome in an arthritis model of K/BxN serum transfer in mice (Kronke et al., 2009). In corroboration, the overexpression of 15-lipoxigenase reduces inflammation, tissue damage, and increases SPM levels (Serhan et al., 2003). Those evidence contributed to the advances in lipidomic research in the last decade, and useful methodologies have been placed to identify and determine levels of lipid mediators in serum or synovial fluid, paving new paths toward understanding its physiological role (Giera et al., 2012). In fact, the development of enzyme immunoassay (Hashimoto et al., 2007) and the application of LC-MS/MS (Giera et al., 2012) for SPM or their precursors detection were key steps for the understanding of the role and temporal profiling of lipids in diseases in humans and rodents. They might be also used to stratify patients in different phases of disease or even be used as predictive for drug responsiveness. A recent study highlights that plasma levels of SMP are a potential biomarker for DMARD responsiveness in patients with RA (Gomez et al., 2020). It was found, using supervised machine-learning methodologies, that increased levels of RvD4, 10S,17S-diHDPA, 15R-LXA4, and MaR1 are linked to DMARD (methotrexate, mono or co-therapy) responsiveness in RA patients. If confirmed in a larger clinical study (the comparison in that study was conducted by assessing 36 DMARD responders vs 26 non-responders), these results might provide important insights for clinicians in terms of disease activity, therapy choice and efficacy (Gomez et al., 2020).

**TABLE 2:** SPM and other lipid mediator levels in patients with arthritis and healthy subjects.

Disease	*n*	Medication	Sample	Method	SPM measured	Observations	References
Rheumatoid arthritis	HC – 30 RA – 30	–	Serum	UPLC-MS/MS	MaR1	Patients with active RA have lower levels of MaR1 than healthy controls, or patients with inactive arthritis.	Jin et al., 2018
	HC – 30 RA – 30	–	Serum	UPLC-MS/MS	RvD1	RA patients have lower levels of RvD1 in the serum compared to healthy controls. RvD1 levels is negatively correlated to connective tissue growth factor, which is elevated in the serum of RA patients.	Sun et al., 2020
	HC – 3 RA – 3	–	Serum	LC-MS/MS	Lipidomic	RA patients have a disruption in SPM levels. Lower levels of RvD3, RvD4, RvE3, AT- LXA_4_, and PGD_2_, and high levels of TxB_2_.	Arnardottir et al., 2016
Rheumatoid arthritis/osteoarthritis	RA – 30	Prednisolone (90%) NSAIDs (83%) Aspirin (13%)	Synovial fluid	ELISA	LXA_4_, 15-epi- LXA_4_, PGE_2_, and LTB_4_	OA patients have lower levels of LXA_4_, 15-epi-LXA_4_, mRNA expression of ALX/FPR2, and 15-LOX, compared to RA patients.	Hashimoto et al., 2007
	OA – 15	Prednisolone (0%) NSAIDs (6%) Aspirin (1%) Statin (1%)					
	RA – 18 OA – 26	NSAIDs (27%) Prednisolone (50%) Other (23%) NSAIDs (70%) Acetaminophen (30%)	Synovial fluid	ESI-MS	Lipidomic	OA patients have lower levels of PD1, LXA_4_, and LXB_4_. RA patients show higher levels of LTB_4_, and LTB_5_. Patients under treatment with NSAIDs, in particular Loxoprofen have higher levels of PGE_2_ when compared to patients taking celecoxib.	Sano et al., 2020
Osteoarthritis	HC – 52 OA – 62	–	Serum	LC-MS/MS	Lipidomic	No changes in E-series and D-series SPMs between groups. Thermic pain is associated with the levels of the precursor 17-HDHA.	Valdes et al., 2017

*17-HDHA, 17-hydroxy docosahexaenoic acid; AT-LXA_4_, aspirin-trigged lipoxin A_4_; ELSA, enzyme-linked immunosorbent assay; ESI-MS, electrospray ionization-tandem mass spectrometry; HC, Healthy control; LC-MS/MS, liquid chromatography– tandem mass spectrometry; 15-LOX, 15-lipoxygenase; LTB_4_, leukotriene B_4_, LXB_4_, lipoxin B_4_; MaR1, maresin 1; NSAIDs, non-steroidal anti-inflammatory drugs; OA, osteoarthritis; PD1, protectin D1; PGD_2_, prostaglandin D_2_, PGE_2_, prostaglandin E_2_, RA: Rheumatoid arthritis; RvD1, resolvin D1, RvD3, resolvin D3; RvD4, resolvin D4; RvE3, resolvin E3; and UPLC-MS/MS, ultra-performance liquid chromatography-tandem mass spectrometry.*

Compelling evidence have shown, in fact, that there is a sharp edge between arthritis disease status and lipid mediator levels (Hashimoto et al., 2007; Arnardottir et al., 2016; Jin et al., 2018; Sano et al., 2020; Sun et al., 2020; Table 2). A disbalance in SPMs correlates with the disease aggressiveness or pathogenesis, indicating SPMs might control disease status (Arnardottir et al., 2016). In patients with active RA, lower plasma levels of MaR1 are found when compared to patients with inactive RA or the healthy control (Jin et al., 2018). Similarly, the levels of RvD1 are decreased in the serum of RA patients and negatively correlated with the connective tissue growth factor (CTGF), which have increased levels in RA patients (Sun et al., 2020). In a lipidomic study, serum levels of SPMs are disrupted in patients with RA when compared to healthy controls, levels of RvD3, RvD4, RvE3, 15-epi-LXA_4_ (AT- LXA_4_), and PGD_2_ in healthy controls are increased, whereas in RA patients higher TxB_2_ levels were found (Arnardottir et al., 2016). This set of data indicate that higher levels of circulating SPMs are found in healthy controls when compared to RA patients (Figure 1). In the synovial fluid, however, patients with OA (usually used as negative controls in studies with RA patients) have lower levels of SPMs or pro-resolving precursors when compared to RA patients (Hashimoto et al., 2007). Specifically, lower levels of LXA_4_, AT-LXA_4_, ALX/FPR2 mRNA, and 15-LOX, were observed in comparison to RA patients (Hashimoto et al., 2007). Of interest, no heathy subjects were considered in that study and despite patients under treatment were considered, no drastic medication influences were noted (Hashimoto et al., 2007). In a similar comparison study, in spite of RA patients have higher levels of SPMs (PD1, LXA_4_, and LXB_4_) when compared to OA, the levels of LTB_4_, and LTB_5_ were also higher in RA patients (Sano et al., 2020). Furthermore, patients under treatment with NSAIDs, in particular loxoprofen, have higher levels of PGE_2_ in comparison to patients taking celecoxib (Sano et al., 2020). These data indicate that while circulating SPM levels are reduced in RA patients (when compared to healthy controls), in the inflammatory foci (joint synovial fluid) higher levels of these lipid mediators can be found (when compared to OA patients; Figure 1).

Importantly, not only the levels of SPMs are effective in reducing disease symptoms, but also the precursors of those molecules might be related to analgesic and anti-inflammatory outcome. In a cohort study, higher levels of 17-HDHA is negatively correlated to thermic pain in patients with OA and healthy controls, whereas there are no changes in E- or D-series SPM levels (Valdes et al., 2017). These shed light in the fact that the whole metabolism of polyunsaturated fatty acids and their metabolites play a crucial role in the disease establishment, progression, and control. For instance, in patients with different forms of arthritis the supplementation with *n*-3 long chain polyunsaturated fatty acids (*n*-3FA) increases the levels of SPM, which negatively correlates with pain score (Barden et al., 2016). This strong evidence demonstrates the endogenous role of SPMs and the importance of these mediators to arthritis. However, there are still several limitations regarding to temporal determination of SPMs in the disease course in human patients.

### SPM Levels and Arthritis Status in Animal Models

There are several models (acute and chronic) used to study the pathogenesis of arthritis as well as the mechanisms of novel anti-inflammatory and anti-rheumatic compounds. Table 3 summarizes the best-known models to study different forms of arthritis with the human symptomatic features phenocopied by them. Serum-transfer of K/BxN mice induces polyarthritis with a T- and B cell-independent component and a major role of neutrophils (Monach et al., 2007). This mouse strain expresses the T cell receptor (TCR) transgene KRN and MHC class II molecule A(g7) and spontaneously develop inflammatory arthritis alongside high titers of autoantibodies to glucose-6-phosphate isomerase (Monach et al., 2007). Transfer of serum or anti-glucose-6-phosphate isomerase antibodies from K/BxN mice into wild-type mice induces polyarthritis (Monach et al., 2007). By contrast, active immunization induces adaptive immunity responsible to the development of polyarthritis in collagen-induced arthritis (CIA) model, which is initiated through intradermal immunization with type II collagen emulsified in complete Freund’s adjuvant (CFA; Courtenay et al., 1980; Asquith et al., 2009). Another polyarthritis model, the collagen antibody-induced arthritis model is induced by the passive transfer of antibodies, in this case a cocktail of monoclonal antibodies that are directed against conserved autoantigenic epitopes in type II collagen, followed by injection of LPS (Terato et al., 1992; Asquith et al., 2009). The antigen-induced arthritis model is another one that is induced by an immunization of the animal. Ovalbumin or, mainly, methylated bovine serum albumin (mBSA) mixed in CFA are the most frequently used antigens. Monoarthritis is induced after a local challenge (usually the knee joint is chosen) with the same antigen (Verri et al., 2008, 2010). Septic arthritis is highly aggressive and with rapidly progression type of infectious arthritis. *Staphylococcus aureus* is the most common causative agent of septic arthritis. Knee joint injection of *S. aureus* induces chronic pain and extensive joint damage and can be used to study this type of infectious arthritis (Staurengo-Ferrari et al., 2017, 2018). Intravenous injection of the bacteria is also used to induce transient bacteremia or sepsis with a percentage of animals developing septic arthritis (Jarneborn et al., 2020; Sultana and Bishayi, 2020). However, the induction of sepsis due to systemic bacterial injection might be a confounder for studying the analgesic effect of new compounds.

**TABLE 3:** Animal models of arthritis.

Duration	Model	Stimulus (route)/Previous immunization	Mono or polyarthritis	Human phenocopied symptoms	References
Acute	Gout	MSU crystals (intra-articular, knee joint)	Monoarthritis	Increased pro-inflammatory cytokine production in the knee joint Synovial inflammation Pain	Amaral et al., 2012
	Zymosan	Zymosan (intra-articular, knee joint)	Monoarthritis		Guerrero et al., 2006
	LPS	LPS (intra-articular, knee joint)	Monoarthritis		Chen et al., 2005; Guerrero et al., 2016
Chronic	AIA	mBSA (intra-articular, knee joint)/immunization with CFA	Monoarthritis	Adaptive and innate component Cartilage destruction Increased cytokine production in the knee joint Synovial inflammation	Verri et al., 2010; Bersellini Farinotti et al., 2019
	CAIA	Cocktail of monoclonal antibodies (intravenous)	Polyarthritis	Adaptive and innate component Antibodies against cartilage epitopes Cartilage destruction Chronic synovial inflammation Increased cytokine production in the knee joint	Terato et al., 1992; Nandakumar et al., 2003
	CIA	Type II collagen (intravenous)/immunization with CFA	Polyarthritis	Adaptive immune component Antibodies against Bone and cartilage destruction Chronic synovial inflammation Joint-specific epitopes Synovial inflammation	Courtenay et al., 1980; Inglis et al., 2007
	K/BxN	Serum transfer containing anti-GPI antibodies (intraperitoneal)	Polyarthritis	Cartilage and bone destruction Innate component Pain Synovial inflammation	Jonsson et al., 2005; Monach et al., 2007
	Prosthesis-related	TiO_2_ (intra-articular, knee joint)	Monoarthritis	Cartilage destruction Increased cytokine production in the knee joint Innate component Pain Synovial inflammation	Borghi et al., 2018
	Septic arthritis	*Staphylococcus aureus* (intra-articular, knee joint)	Monoarthritis	Bacterial growth and spread Cartilage and bone destruction Chronic synovial inflammation Pain Synovial inflammation	Staurengo-Ferrari et al., 2018
		*Staphylococcus aureus* (intravenous)	Polyarthritis		Fatima et al., 2017; Jarneborn et al., 2020; Sultana and Bishayi, 2020

*AIA, adjuvant-induced arthritis; CAIA, collagen antibody-induced arthritis; CFA, complete Freund adjuvant; CIA, collagen-induced arthritis, LPS, lipopolysaccharide; MSU, monosodium urate; and TiO_2_, titanium dioxide.*

In mice, a temporal regulation of lipid mediators was also recently established (Arnardottir et al., 2016). This study demonstrates the resolution phase lipid class switch, and more importantly, the role of the SPMs in arthritis resolution and regulation (Arnardottir et al., 2016; Figure 2). In the K/BxN RA model, local joint inflammation starts after the second serum injection, and is accompanied with joint leukocyte recruitment, redness, and edema. Because this is a self-resolving model, 11 days after serum challenge, the resolution phase starts with the reduction of inflammation and clinical score (Arnardottir et al., 2016). Lipidomic analysis reveals that after challenge, an increase in pro-inflammatory eicosanoids and concomitant decrease in most SPMs is observed (Arnardottir et al., 2016). In fact, as clinical manifestation starts, the levels of PGE_2_, PGD_2_, TXB_2_, and LTB_4_ increases in the paw tissue. Interestingly, 16 days after challenge, a robust lipid mediator class switch restores the levels of SPMs found in the naïve animals, healthy controls. In the resolution phase, D-series Rv (such as RvD1, RvD2, RvD3, and RvD4), MaR1, and PD1, have their levels restored (Arnardottir et al., 2016). Moreover, a second K/BxN serum challenge disrupts resolution phase, maintains the clinical score, and levels of pro-inflammatory lipid mediators, limiting the temporal class switch and delaying resolution (Figure 2).

In conclusion, pre-clinical and clinical data (Table 2) demonstrate that the fluctuation in SPMs levels has an inverse correlation to arthritis status and associated symptoms. Therefore, boosting SPM levels might be a potential approach for the treatments of rheumatic diseases.

## Endogenous SPM’s Role and Levels in Arthritis Guiding Possible Therapeutic Approaches

The contemporary understanding of the inflammation resolution process transformed the field and opened novel avenues to a diverse niche of therapeutic possibilities. Also, the publication of web-based resource named as Atlas of Inflammation Resolution (Serhan et al., 2020) is also an important step toward elucidating the process during the resolution process. This website provides a molecular interaction map that allows users to visualize molecular pathways relevant to inflammation and its resolution. These tools might allow a better understanding of the resolution process and the potential uses and properties of SPMs. The endogenous SPMs play important roles in the course of rheumatic diseases, as described above. Endogenous SPMs are effective in limiting inflammation, protecting joint damage, therefore decreasing arthritis pain. In fact, supplementation with omega-3 fatty acids or SPM precursors have shown efficacy in clinical trials (Fattori et al., 2020). Altogether, those facts elicited the possibilities of SPM exogenous administration in therapeutic approaches. In fact, there were attempts to test clinically the role of RvE1 in the dry eye syndrome (clinicaltrials.gov identifier: NCT01675570, NCT00799552). Despite the completion of the clinical studies, up to this point, no final results were published. Focusing on pain, a major symptom in rheumatic conditions, evidence have shown that SPMs can block nociceptor activation, hence decreasing pain (revised, Fattori et al., 2019, 2020; Allen et al., 2020).

Although peripheral inflammation fluctuates between asymptomatic and symptomatic periods, the pain is still commonly persistent between those phases (Allen et al., 2020). In the K/BxN-induced self-resolving RA model, even after resolution of joint inflammation, pain is persistent (Rifbjerg-Madsen et al., 2017; Allen et al., 2020). By establishing the lipid profile in the DRG neurons (where the cell body of joint-innervated nociceptors are located), Allen and colleagues show that after arthritis resolution there is a decrease in the levels of MaR1 in the DRG. This is followed by M1 macrophage infiltration in the DRG and cytokine production (IL-1β and TNF-α) leading to neuronal activation and hyperalgesia. Treatment with MaR1 reduces mechanical hyperalgesia, DRG M1 macrophage infiltration, and decreases cytokine expression in a receptor-dependent manner (Allen et al., 2020). Similarly, persistent hyperalgesia is also observed after diseases resolution in the animal model of mBSA antigen-induced monoarthritis (Goncalves et al., 2019). This phenotype is due to elevated levels of TNF-α in the knee joint-innervated DRG, which might possibly be modulated by pro-resolving molecules. Although limited, the literature supports the important role of endogenous SPMs in the control of arthritis. Furthermore, these findings suggest the use of SPMs in therapeutic approaches, and its suitability for the development of novel treatments to arthritis (Figure 2). Thus, in this section, we focus on preclinical data showing the endogenous physiopathological role and therapeutic potential of SPMs in arthritis-driven inflammation and pain. The pro-resolving properties are described based in the precursor molecule, i.e., AA, DPA, DHA, or EPA. Table 4 summarizes our discussion.

**TABLE 4:** Pre-clinical effects of SPMs in rheumatic conditions.

	Mediator	Species	Model/Stimulus	Dose or concentration	Route	Outcome	References
AA	14,15-EET	Mouse	Ovariectomy-induced bone loss	17 mg/kg	i.p.	Inhibits osteoclastogenesis and bone loss. Decreases RANKL:OPG ratio and inflammatory cytokines	Guan et al., 2015
		*In vitro* – Raw264.7 macrophages	RANKL	2 μM	–	Suppresses RANKL-induced osteoclast differentiation, phosphorylation of NF-kB, ERK, and JNK. Prevents the production of reactive oxygen species.	
	EC1728 (sEHi – EET indirect)	Dog	Self-occurring arthritis	5 mg/kg	p.o.	Decreases pain.	McReynolds et al., 2019
	ETT mixture	*In vitro* – canine chondrocytes	IL-1β	0.4 μg/mL	–	Increases cell viability and decreases levels of TNF-a and IL-6.	
	TPPU (sEHi – EET indirect)	Mouse	CIA	10 mg/kg	p.o.	Ameliorates hyperalgesia, histopathological score, and cartilage destruction. Decreases Th1- and Th17-related cytokine levels and increases Treg-related ones.	Trindade-da-Silva et al., 2020
	PGD_2_	Mouse	CIA	0.6 mg/kg	i.pl.	Reduces immune cell infiltration, bone erosion, and arthritis incidence.	Maicas et al., 2012
	15d-PGJ2	Rat	CIA	10 μg/kg	i.p.	Decreases paw volume, arthritis score, mononuclear cell infiltration, and pannus invasion.	Kawahito et al., 2000
		Mouse	CIA	30 μg/kg	i.p.	Decreases clinical, histopathological and radiographic scores, edema, and lipid peroxidation.	Cuzzocrea et al., 2002
		*In vitro* – human synovial fibroblasts	TNF-α	1–3 μM	–	Reduces MMP-13 expression and NF-kB activation.	Lin et al., 2011
		*In vitro* – human osteoarthritic chondrocytes	IL-1β	10 μM	–	Blocks PGE_2_ synthesis, and partially reduces the expression of COX-2.	Fahmi et al., 2002
	15d-PGJ2 nanocapsule	Mouse	Gouty arthritis/MSU crystals	30 μg/kg	s.c.	Reduces mechanical hyperalgesia, edema, leucocyte recruitment, oxidative stress, pro-inflammatory cytokines, and mRNA expression of NLRP3 inflammasome components. Increases levels of IL-10.	Ruiz-Miyazawa et al., 2018
		*In vitro* – bone marrow-derived macrophages.	MSU crystal	3 μM	–	Decreases IL-1β release.	
	LXA_4_	Mouse	Zymosan-induced	20 ng	i.a.	Reduces edema and leucocyte recruitment.	Conte et al., 2010
DPA/DHA	RvD1	*In vitro* – human osteoarthritic chondrocytes.	IL-1β	10 μM	–	Suppress COX-2, iNOS, and MMP-13 expression. Reduces PGE2 and NO levels.	Benabdoune et al., 2016
			HNE	10 μM	–	Reduces apoptosis, caspase-3 activation and lactate LDH release. Increases the levels of Bcl2, AKT, and GSH.	
		Mouse	CIA	100 ng	i.v.	Decreases angiogenesis and CTGF levels. Increases miRNA-146a-5p expression.	Sun et al., 2020
		*In vitro* – fibroblast-like synoviocyte	–	100 nM	–	Decreases the expression of pro-inflammatory cytokines and CTGF by upregulating the expression of miRNA-146a-5p and inhibiting STAT3 activation.	
		Mouse	CIA	500 ng	i.p.	Attenuates clinical score, cartilage degradation, and bone resorption. Decreases synovial proliferation, serum markers of cartilage and bone damage, and inflammatory mediators.	Benabdoun et al., 2019
		*In vitro –* Raw264.7	LPS M-CSF RANK-L	500 nM	–	Reduces osteoclast differentiation, expression of inflammatory mediators, and bone erosion.	
	AT-RvD1	Rat	CFA-induced arthritis	100 ng	i.p.	Decreases mechanical hyperalgesia and levels of pro-inflammatory cytokines (TNF-α and IL-1β).	Lima-Garcia et al., 2011
	RvD3	Mouse	K/BxN serum	100 ng	i.p.	Decreases arthritis clinical score, edema, and leucocyte recruitment. Reduces local eicosanoid levels (e.g., LTB_4_, PGE_2_, and TXB_2_) in an ALX/FPR2-deperndent manner.	Arnardottir et al., 2016
	RvD5	Mouse	K/BxN serum	150 ng	i.p.	Reduces arthritis clinical score, edema, arthritis-induced weight loss, and levels of prostaglandin and LTB_4_. The effects were dependent of GPR101 receptor.	Flak et al., 2020
	MaR1	Mouse	K/BxN serum	100 ng	i.p.	Attenuates mechanical hypersensitivity; reduces monocyte/macrophage infiltration in the DRG.	Allen et al., 2020
		Mouse	CIA	100 ng	i.v.	Reduces arthritis clinical score, and pro-inflammatory cytokine levels (TNF-α, IL-β, IL-6, IFN-γ, IL-17A). Increases levels of IL-10, TGF-β, and miR-21. Regulates Treg/Th17 balance.	Jin et al., 2018
EPA	RvE1	*In vitro* – Raw264.7	RANKL	100 nM	–	Reduces osteoclast differentiation, bone resorption, and expression of osteoclast-related genes. Decreases IL-17-induced expression of RANKL, COX-2 mRNA, and synthesis of PGE_2_.	Funaki et al., 2018

*AA, arachidonic acid; AKT, protein kinase B; AT-RvD1, aspirin-trigged Resolvin D1; Bcl2, B-cell lymphoma 2; EET, epoxyeicosatrienoic acid; 15d-PGJ_2_, 15-deoxy-delta-12,14-prostaglandin J_2_; CIA, collagen-induced arthritis; COX-2, cyclooxygenase-2; CTGF, connective tissue growth factor; DHA, docosahexaenoic Acid; DPA, docosapentaenoic acid; DRG, dorsal root ganglia; ERK, extracellular-signal-regulated kinase; GSH, reduced glutathione; HNE, 4-hydroxy-2-non-enal; i.a., intra-articular; i.p., intraperitoneal; i.pl., intraplatar; i.v. intravenous; IFN-γ, interferon-gamma; IL-10, interleukin-10; IL-17, interleukin-17; IL-1β, interleukin-1β; IL-6, interleukin-6; iNOS, inducible nitric oxide synthase; JNK, c-Jun N-terminal kinase; LDH, lactate dehydrogenase; LPS, lipopolysaccharide; LTB4, leukotriene B4; M-CSF, macrophage colony-stimulating factor; MaR1, maresin 1; MMP-13, matrix metalloproteinase-13; MSU, monosodium urate; NF-κB, nuclear factor-κB; NLRP3, NLR Family Pyrin Domain Containing 3; NO, nitric oxide; OPG, osteoprotegerin; p.o., per oral; PGD_2_, prostaglandin D_2_; PGE_2_, prostaglandin E_2_; RANKL, receptor activator of nuclear factor-kappa B ligand; RvD1, resolvin D1, RvD3, resolvin D3; RvD5, resolvin D5; RvE1, resolvin E1; s.c., subcutaneously; sEHi, soluble epoxy hydrolase; STAT3, signal transducer and activator of transcription 3; TGF- β, transforming growth factor beta; TNF-α, tumor necrosis factor-α; TPPU, N-[1-(1-oxopropyl)-4-piperidinyl]-N’-[4-(trifluoromethoxy)phenyl)-urea; and TXB_2_, thromboxane B_2_.*

### AA-Derived SPMs

The AA is rapidly correlated to inflammation and fever. However, under the action of different enzymes, AA is metabolized in lipids with anti-inflammatory, analgesic, and pro-resolutive proprieties, such as, epoxyeicosatrienoic acids (EETs; Gilroy et al., 2016), prostaglandin D_2_ (PGD_2_), 15deoxy-Δ^12,14^-prostaglandin J2 (15d-PGJ_2_), and lipoxins.

Epoxyeicosatrienoic acids are precursors for several SPMs (Schmelzer et al., 2005), including lipoxins. Administration of 14,15-EET inhibits bone resorption and osteoclastogenesis in ovariectomy-induced bone loss in rats. In addition, treatment with it decreases RANKL:OPG ratio and inflammatory cytokine. *In vitro*, 14,15-EET treatment in RAW264.7 and bone marrow mononuclear cells, suppresses RANKL-induced osteoclast differentiation, and reduces the activation of several osteoclastogenesis pathways. Altogether, the data points to this lipid as possible therapeutic strategy for osteoclast-related disorders, such as arthritis (Guan et al., 2015).

In spite the potent effects, ETTs are rapidly metabolized by soluble epoxy hydrolase (sEH) in ineffective molecules. Considering the instability of the compound, a therapeutic approach relaying in the use of soluble epoxy hydrolase inhibitors (sEHi), which potentialize endogenous ETT effects and SPM synthesis, can be useful. Interestingly, the administration of ETT, or sEHi have similar effects in suppressing RANKL-induced osteoclast (Guan et al., 2015). In dogs with self-occurring OA, pain was reduced upon administration of the sEHi EC1728 (McReynolds et al., 2019). In addition, the administration of a mixture of EETs to canine chondrocytes increases cell viability and reduces IL-6 and TNF-α expression. In another study, the effects of daily treatment with the sEHi 1-trifluoromethoxyphenyl-3-(1-propionylpiperidin-4-yl) urea (TPPU) ameliorates mechanical hyperalgesia, edema, histopathological score, and cartilage destruction in the CIA model. In addition, the administration of TPPU reduces Th1- and Th17-related pro-inflammatory cytokines, while increasing Treg cells cytokine profile expression (Trindade-da-Silva et al., 2020), demonstrating the therapeutic potential and immunoresolvent proprieties of ETTs in arthritis.

Downstream AA metabolization, prostanoids are a major group comprehending for example, prostaglandin E_2_ (PGE_2_), prostaglandin D_2_ (PGD_2_), and its degradation product, the 15-deoxy-Δ^12,14^-PGJ2 (15d-PGJ2). PGD_2_ has been described to have a complex role in inflammation, with pro- and anti-inflammatory effects in differences circumstances (Ajuebor et al., 2000). In a model of CIA, the levels of PGD_2_ increase in a time-dependent manner, accompanied by the increase in the expression levels of its metabolization enzymes (hematopoietic PGD synthase and lipocalin-type PGD synthase), and its receptors (PD1 and PD; Maicas et al., 2012). In corroboration, the administration of PD1 antagonist (MK0524) augments arthritis incidence and severity, increases levels of IL-1β, CXCL-1, and PGE_2_, whereas reduces the levels of IL-10. On the other hand, the administration of PGD_2_, or PD1 agonist (BW245C) significantly reduce arthritis incidence, inflammation response and joint damage, indicating the contribution of anti-inflammatory AA metabolites to the control of pain.

15deoxy-Δ^12,14^-prostaglandin J2 is a natural and potent agonist of peroxisome proliferator–activated receptor γ (PPARγ) and is well described to have anti-inflammatory actions (Rajakariar et al., 2007; Li et al., 2019). In the arthritis perspective, 15d-PGJ_2_ ameliorates the outcome of CIA in rats by decreasing paw volume, arthritis score, mononuclear cell infiltration, and pannus invasion (Kawahito et al., 2000). In a similar study, the cyclopentenone administration also reduces CIA clinical, histopathological and radiographic scores, edema, and CIA-induced lipid peroxidation (Cuzzocrea et al., 2002). In human synovial fibroblast, the treatment with 15d-PGJ_2_ reduces TNF-α-induced matrix mieloproteinase-13 expression in culture. While an agonist of PPARγ the lipid inhibits NF-κB translocation to nucleus in a PPARγ-independent manner by acting on IKK activation, indicating it can promote anti-inflammatory effect by targeting multi-pathways (Lin et al., 2011). In human osteoarthritic chondrocytes, treatment with 15d-PGJ_2_ blocks PGE_2_ production with a milder effect on COX-2 expression. Interestingly, in the presence of 15d-PGJ_2_ independent of a COX-2 inducer, the enzyme levels are increased (Fahmi et al., 2002). Interestingly, pharmaceutical formulation with 15d-PGJ_2_ have proven to be even more effective (Alves et al., 2011; Clemente-Napimoga et al., 2012; Napimoga et al., 2012; Ruiz-Miyazawa et al., 2018). Nanocapsules loaded with 15d-PGJ_2_ ameliorate MSU-induced pain and inflammation in a model of gouty arthritis. The treatment reduces mechanical hyperalgesia, edema, leucocyte recruitment to the knee joint, pro-inflammatory cytokines, and oxidative stress. The effects were all PPARγ-dependent (Ruiz-Miyazawa et al., 2018). These data support that 15d-PGJ_2_ resolves inflammation by acting in different pathways in relevant cells in the context of arthritis.

The lipoxins are the first described SPMs (Serhan et al., 1984), and the first lipid mediators of the endogenous anti-inflammatory and resolutive machinery (Serhan, 2005). In fact, in a model of CIA, the levels of Lipoxin A_4_ (LXA_4_) increase according to diseases progression achieving its peak on the resolution phase onset (Chan and Moore, 2010). Although, endogenous levels are important, the treatment with LXA_4_ has a protective effect in a model of zymosan-induced arthritis (Conte et al., 2010). Local treatment with LXA_4_ reduces edema and leucocyte recruitment in a receptor-dependent manner as treatment with BOC-1, an ALX/FPR2 receptor antagonist, abrogates LXA_4_ effects. In addition, treatment with BML-111, a potent ALX/FPR2 agonist, also decreases immune cell recruitment to the knee joint (Zhang et al., 2008; Conte et al., 2010). Complementarily, the administration of aspirin increases the levels of lipoxins (i.e., AT-LXA_4_), thus reducing the inflammation also in a receptor-dependent fashion. The potent anti-inflammatory and analgesic effects of AA-derived SPMs and their receptor-mediated action allows the development of new formulations and novel drug candidates for the control of arthritis symptoms and progression.

In conclusion, AA-derived SPMs include EETs, prostaglandins, and lipoxins, have been shown to have pro-resolving effects in arthritic-related psychopathological events, by decreasing inflammation, cytokine and chemokine levels, osteoclast activation, bone degradation, and importantly reducing arthritis-related pain.

### DPA/DHA-Derived SPMs

The D-serie lipid mediators comprehend Rvs, MaRs, and PDs, which have been shown to act in the reduction of inflammation, activation of non-phlogistic macrophages, or direct blockage of neuronal activity (Serhan, 2014). In arthritis, the effect of RvD1, AT-RvD1, RvD3, RvD5, and MaR1 were tested in animal models.

In arthritis, the persistent inflammation could lead to extended joint destruction, bone resorption, and function loss (Scott et al., 2010). As such, the relevance of RvD1, an agonist of GPR32 and ALX/FPR, was primarily described by its *in vitro* action in human osteoarthritic chondrocytes (Benabdoune et al., 2016). Treatment with it suppresses IL-1β-induced expression of COX-2, iNOS, and metalloproteinase-13, which results in lower levels of PGE_2_ and nitric oxide (NO) production accompanied by the reduction in of NF-κB-p65, p38/MAPK, and JNK phosphorylation (Benabdoune et al., 2016). In addition to act reducing inflammation, RvD1 also reduces 4-hydroxynon-enal-induced apoptosis, suppressing caspase-3 activation and lactate dehydrogenase (LDH) release and increases Bcl2, AKT, and GSH (Benabdoune et al., 2016). RvD1 reduces the differentiation of macrophages in osteoclasts inhibiting the expression of tartrate resistant acid phosphatase and cathepsin-K (Benabdoun et al., 2019). Treatment with it also decreases the expression of inflammatory molecules such as, TNF-a, RANK, and PGE_2_, and increases IL-10 (Benabdoun et al., 2019). In summary, the data indicate that *in vitro* experiments in the context of arthritis, RvD1 reduces inflammatory pathways while increase anti-apoptotic ones, giving confidence for a novel *in vivo* therapeutic approach.

*In vivo*, RvD1 reduces CIA clinical score, inflammation, bone and joint destructions (Benabdoun et al., 2019) while other study show that this SPM reduces angiogenesis and decreases the expression of CTGF, and levels of IL-6, TNF-α, and IL-1β (Benabdoun et al., 2019; Sun et al., 2020). Using fibroblast-like synoviocyte culture, treatment with RvD1 lowers the levels of CTGF and reduces the cellular proliferation. Mechanistically, RvD1 upregulates the expression of miRNA-146a-5p, which was proven to decrease the levels of inflammatory molecules and CTGF, *in vivo* and *in vitro* via the inhibition of STAT3 activation (Sun et al., 2020). Altogether, this data shed light in the protective effect of RvD1 to arthritis progression and related bone disorders (Benabdoun et al., 2019). The systemic delivery of aspirin-trigged analog, AT-RvD1, or 17(R)-RvD1 is also described to reduced CFA-induced arthritis hyperalgesia and cytokines expression (TNF-α and IL-1β; Lima-Garcia et al., 2011).

RvD3 is another GPR32 and ALX/FPR agonist that is also described to resolve preclinical arthritis (Arnardottir et al., 2016). In the K/BxN arthritis model, treatment with RvD3 decreases the arthritis clinical score, edema, and leucocyte recruitment. Importantly, the exogenous administration, was also effective to reduce the levels of LTB_4_, PGE_2_, PGD_2_, PGF_2α_, TXB_2_, and 8-iso-PGF_2α_. In animals lacking ALX/FPR2/3 receptor, the effects of RvD3 were abolished, indicating its effects are dependent on that receptor (Arnardottir et al., 2016). Similarly, RvD5 reduces prostaglandin and LTB_4_ levels and improves arthritis clinical score, edema and weight loss, by signaling via its receptor GPR101 in the K/BxN model (Flak et al., 2020). However, an important point regarding RvD5 is the demonstration of its lack of analgesic effect in female mouse (Luo et al., 2019). Considering the higher incidence of RA in women, treatment with RvD5 would have this drawback compared to other analgesic SPMs.

Compelling evidence have shown that MaR1, an agonist of LGR6 receptor (Chiang et al., 2019), have analgesic, anti-inflammatory, and neuroprotective proprieties (Gao et al., 2018; Fattori et al., 2019; Yang et al., 2019; Wang et al., 2020). In the K/BxN model, lower endogenous levels of MaR1 in the DRG correlates with persistent mechanical hyperalgesia and systemic delivery of it, either before or after the clinical signs of self-resolution, significatively reduces mechanical hyperalgesia (Allen et al., 2020). In addition, MaR1 inhibits macrophage recruitment to the DRG and reduces the population of M1 macrophages, contributing to pain reduction (Allen et al., 2020). Regarding its effect on neurons, previous work of our group demonstrated that MaR1 reduces CGRP release in cultured DRG and TRPV1 activation-induced calcium influx *in vivo* (Fattori et al., 2019). By this neuronal regulation, intrathecal treatment with MaR1 reduces the peripheral recruitment of inflammatory neutrophils and macrophages, reducing in addition to pain, peripheral inflammation (Fattori et al., 2019). In corroboration, Allen et al. (2020) demonstrated that MaR1 also reduces calcium influx induced by capsaicin (TRPV1 agonist) in a GPCR-dependent manner and CGRP release. In another study, CIA-associate clinical score and cytokine profile were abrogated with MaR1 treatment (Jin et al., 2018). The administration of MaR1 reduces the levels of TNF-α, IL-β, IL-6, IFN-γ, IL-17, and increases the levels of IL-10 and TGF-β. Further, the number of Th17 cells were reduced, whereas the number of Treg cells were increase with the treatment. There are strong evidence that RA patients have increased number of Th17 cells and decreased proportion of Treg cells (Niu et al., 2012; Samson et al., 2012). The unbalance in Treg/Th17 ratio contributes to triggering autoimmunity and inducing inflammation (Niu et al., 2012). In terms of mechanism, treatment with MaR1 induces the overexpression of miR-21, which increases Treg cell number and restore Treg/Th17 balance, therefore improving disease outcome (Jin et al., 2018).

In addition to the isolated D-serie SPMs biologic activity, here reviewed, metabolomic pathways also generate pro-resolving sulfido-conjugates, which are effective in elicit pro-resolving phagocyte functions and tissue regeneration (Chiang et al., 2018; de la Rosa et al., 2018; Jouvene et al., 2019; Levy et al., 2020). Despite the effect that MaRs-, PDs-, or Rvs-conjugates in tissue regenerations were not explored in rheumatic diseases, this recent described branch of pro-resolving mediators might add to novel approaches for the treatment of arthritis, enlarging clinical perspectives of these classes.

In summary, the presented pre-clinical data highlight the important endogenous role of D-serie Rvs, MaRs, and PDs in the control of arthritis symptoms. The current literature demonstrates a series of protective effects, in which the SPMs orchestrate the resolution of the inflammatory process, by decreasing the expression and/or release of pro-inflammatory cytokines, chemokines, and lipid mediators. Furthermore, *in vitro* or *in vivo* treatment diminish the differentiation and activation of osteoclasts, which decrease bone degradation. Combined, the pro-resolutive, protective, immunoresolvent and analgesic properties of D-serie SPMs improve the overall disease outcome.

### EPA-Derived SPMs

The E-serie resolvins include RvE1, RvE2, and RvE3. While their anti-inflammatory effects have been demonstrated in models of asthma (Siddiquee et al., 2019), atherogenesis (Hasturk et al., 2015), and dry eye syndrome (Li et al., 2010), and analgesic effects in models of inflammatory pain (Xu et al., 2010); these mediators have not been tested in rheumatic disease models. *In vitro*, however, treatment with RvE1 suppresses RANK-L-induced RAW264.7 differentiation in osteoclasts, reduces the expression of osteoclast-related genes, and decreases the process of bone reabsorption. Furthermore, RvE1 inhibits IL-17-induced expression of RANK-L, COX-2, and PGE_2_ synthesis. These data indicate that RvE1 act on pathways that interfere with osteoclast differentiation and bone reabsorption, which might indicate that E-serie Rv could be effective in reducing arthritis symptoms. However, it remains to be determined whether *in vivo* effects during arthritis are observed with any of the RvE.

## Conclusion and Future Perspectives

Focusing on the therapeutic point of view, we envision at least 4 different SPM-based therapeutic approaches based on their pathophysiological roles in arthritis: (1) Development of more stable SPM analogs. BML-111 (a LXA4 analog) and benzo-diacetylenic-17R-RvD1-methyl ester (RvD1 analog) are examples that we can highlight. These compounds are the agonists of ALX/FPR2 and GPR32, respectively. BML-111 has demonstrated activity in two different models of arthritis (Zhang et al., 2008) and therefore the development of novel and stabler analogs might help overcome the issue of SPM half-life. RX-10045 is an example that reached clinical trials. RX-10045 is a pro-drug for a RvE1 analog that has been tested for dry eye syndrome. (2) Development of pharmaceutical formulations to improve the effect of SPMs or their analogs. Since SPM can be rapidly metabolized, the development of systems aiming at protecting or enabling targeted delivery of SPM might be useful. For instance, SPM-loaded nano capsules or ectosomes in addition to the use of polymeric nanoparticles might help overcome this issue. For instance, 17(R)-RvD1- or a lipoxin analog (benzo-LXA4)-loaded ectosomes accelerates wound healing and reduces CFA-induced temporomandibular joint (Norling et al., 2011). Another example is the utilization of an engineered polymeric nanoparticle containing the Ac2-26 (an ANXA1 mimetic peptide). This system has demonstrated efficacy by promoting wound repair in a model of colitis (Leoni et al., 2015). (3) Development of pro-resolving small molecules. Given SPM receptors are G protein-coupled receptors (GPCRs), the discovery of novel orthosteric agonists (binds to the same site as the endogenous ligand), positive allosteric modulators (binds to a different site and increases the activity of the endogenous ligand), or small molecules with the ability of inducing biased agonism (activates a specific signaling pathway of a given GPCR) are also interesting and promising alternative for stimulating resolution pathways. (4) Development of SPM catabolism blockers. Like monoamine oxidase inhibitor drugs used for depression and in some cases chronic pain, the discovery of compounds with the ability of targeting enzymes that promote SPM catabolism might be useful to increase endogenous or exogenous SPM availability and thereby their effect. As we discussed above, evidence supports that disease severity correlates with low levels of SPMs, thus, enhancing their levels might be a manner of improving patient well-being by enhancing endogenous SPMs levels.

Interestingly, SPMs might be also useful for infectious arthritis. If not by bactericidal effect *per se*, SPMs stimulate the phagocytosis and clearance of different pathogens. For instance, RvD1 synergizes with ciprofloxacin to promote the non-phlogistic phagocytosis of *Pseudomonas aeruginosa* during lung infection (Codagnone et al., 2018) and against *Escherichia coli* (Chiang et al., 2012). Similarly, other SPMs such as RvD2 (Spite et al., 2009), MaR1 (Hao et al., 2019), or PDX (Xia et al., 2017) decrease local and systemic bacterial burden, which leads to increased survival in model of sepsis. These effects could be helpful because: (i) new resistant mechanisms are constantly being developed by microorganisms, and therefore, compounds that block pain and actively fight infection are likely to be more effective. (ii) Some antibiotics, such as the β-lactam, induce bacteriolysis releasing LPS and LTA that can induce post-infectious sequalae due persistent activation of immune cells (Ginsburg, 2002). Therefore, in addition to block pain, SPMs enhance bacterial clearance, lower antibiotic requirements, and shorten resolution time interval (Chiang et al., 2012). These effects can contribute to reduce antibiotic resistance and post-infectious sequelae.

Concluding, in the past decades, the advent of SPM characterization allowed the establishment of their pivotal endogenous role in different disease status and symptomatology. There are pre-clinical and clinical evidence that arthritis initiation, symptoms development, and its resolution line up well with the fluctuation in endogenous SPMs levels in a manner that low levels of SPMs correlate with disease severity. In turn, using the reverse approach, treatment with/replenishing/diminishing the metabolization of AA-, DHA/DPA-derived SPMs was demonstrated to improve overall disease outcome, effectively reducing inflammation, pain, and bone destruction. Those lipids reprogram immune cells, block neuronal activity, or modulate the host response, without compromising the immune system or offering undesired side effects. Thus, the understanding of SPMs endogenous roles in arthritis has open novel venues for better understanding the disease itself, therapeutic approaches, and monitoring disease activity and treatment efficacy.

## Author Contributions

All authors contributed significantly to the writing and conception of this review article as well as approved the final version of the manuscript.

## Conflict of Interest

The authors declare that the research was conducted in the absence of any commercial or financial relationships that could be construed as a potential conflict of interest.

## Publisher’s Note

All claims expressed in this article are solely those of the authors and do not necessarily represent those of their affiliated organizations, or those of the publisher, the editors and the reviewers. Any product that may be evaluated in this article, or claim that may be made by its manufacturer, is not guaranteed or endorsed by the publisher.
